# Hospital Isolation in Scarlet Fever

**Published:** 1901-03-02

**Authors:** 


### HOSPITAL ISOLATION IN SCARLET FEVER.

In a very suggestive paper read before the last meeting of the Society of Medical Officers of Health Dr. Millard discussed the influence of hospital isolation in scarlet fever. The thesis which Dr. Millard endeavoured to support may be divided into two parts. First, he showed what is undoubtedly true, namely, that although the great fall in the mortality from scarlet fever which took place about twenty or twenty-five years ago is often attributed to the introduction of isolation hospitals, it really occurred quite independently. The contrast between the fearful mortality from scarlet fever prior to the years 1875-80 and the insignificant death-rate and fatality (irrespective of prevalence) in subsequent years had, said Dr. Millard, been ascribed to the influence of isolation hospitals. But he showed, as Dr. Wilson, of Ayr, had previously done, that this "great slump" in scarlet fever mortality occurred in a large proportion of the towns mentioned before the erection of the hospitals had begun, and that it was equally pronounced where there had never been any such hospitals, and even in rural districts. He showed also that the same fall in the scarlet fever mortality had been observed of recent years in the United States of America, where isolation is unknown. These facts have to be faced, and it is evidently useless for those who advocate the universal enforcement of isolation in infectious maladies to base their proposals on the obvious decrease in the prevalence of scarlet fever which has occurred since the introduction of isolation hospitals, seeing that the same decrease is equally obvious in districts where such hospitals have not yet come into being. In giving prominence to these facts Dr. Millard has done well. He has, however, gone further, and has set up a theory to the effect that the aggregation of cases in isolation hospitals has developed a type of fever of increased persistence and infectivity, of which the "return cases" are the product, and which will in course of time be felt in a generally increased virulence of scarlet fever throughout the land. Clearly this is mere hypothesis, and does nothing to strengthen the important generalisation that the lessened virulence of scarlet fever during recent years has not been, as is often taken for granted, the result of hospital isolation. Are we, then, to throw overboard our hospital system, and revert to the barbaric practices of former times? Certainly not.

What we have to recognise is that in the epidemiology of scarlet fever many factors are engaged, and that the rise and fall of the prevalence of the disease, or of its fatality (two somewhat different matters), are probably the result of the coincidence or co-operation of varying groups of such factors, of the nature of which we know but little. As there can be no doubt, however, that personal infection is one of the chief means by which the disease is spread, it is only fair to believe that the isolation of those affected must be useful so far as it goes; and as there can be no doubt whatever that in the treatment of the poor such hospitals are of great help in saving life, by all means let us retain them and keep up their efficiency in every possible way. But that personal infection is by no means a dominant influence in the epidemiology of scarlet fever, and that isolation plays but a secondary part in restraining its diffusion, is manifest enough. One single instance is sufficient to show that, whatever influence for good hospital isolation may possess, it is overridden by other influences which are at work.

Take, for example, the seasonal variation in the prevalence of scarlet fever: this entirely overrides all that we are able to effect by isolation. At the very moment when the proportion of cases isolated is at the greatest, and when the actual number of centres of infection is at its lowest, the disease begins again to spread; while at the very season when the hospitals cannot take in all the applicants for admission, and when the number of centres of infection remaining unisolated stand at the highest, then the disease begins to die away. This is a sequence of events which occurs year after year, and it shows well how comparatively insignificant is the effort of isolation in the face of epidemic influences of whose nature we still remain very ignorant. While, then, we fully agree with those who would maintain our hospitals in the highest state of efficiency, on the principle that every little helps, we hold with Dr. Millard that the influence of hospitals in controlling the prevalence or in modifying the fatality of scarlet fever is by no means so fully proven as to justify those who would extend compulsory powers and would remove to hospitals cases the surroundings of which offer reasonable facilities for a moderate degree of isolation at home. Hospital isolation is one factor—it can hardly help but be even a considerable factor—in lessening scarlet fever prevalence, but it certainly is not the main factor; and in proportion as this proposition can be maintained the excuse for compulsory hospital isolation of fairly well-to-do patients is diminished, and the propriety of undertaking the care of such patients at the expense of the rates becomes less apparent.

